# Landscape of Adrenal Tumours in Patients with Congenital Adrenal Hyperplasia

**DOI:** 10.3390/biomedicines11113081

**Published:** 2023-11-16

**Authors:** Mara Carsote, Ana-Maria Gheorghe, Claudiu Nistor, Alexandra-Ioana Trandafir, Oana-Claudia Sima, Anca-Pati Cucu, Adrian Ciuche, Eugenia Petrova, Adina Ghemigian

**Affiliations:** 1 Department of Endocrinology, Faculty of Medicine, Carol Davila University of Medicine and Pharmacy, 020021 Bucharest, Romania; carsote_m@hotmail.com; 2 Clinical Endocrinology Department, C.I. Parhon National Institute of Endocrinology, 020021 Bucharest, Romania; alexandratrandafir26@gmail.com (A.-I.T.); oanaclaudia1@yahoo.com (O.-C.S.); jekined@yahoo.com (E.P.); adinaghemi@yahoo.com (A.G.); 3 Ph.D. Doctoral School of Carol Davila, University of Medicine and Pharmacy, 020021 Bucharest, Romania; ancutapati@gmail.com; 4 Department 4—Cardio-Thoracic Pathology, Thoracic Surgery II Discipline, Faculty of Medicine, Carol Davila University of Medicine and Pharmacy, 020021 Bucharest, Romania; adrianciuche@gmail.com; 5 Thoracic Surgery Department, “Dr. Carol Davila” Central Emergency University Military Hospital, 020021 Bucharest, Romania; 6 Department of Endocrinology, Faculty of Midwifery and Nursing, Carol Davila University of Medicine and Pharmacy, 020021 Bucharest, Romania

**Keywords:** adrenal tumour, adrenal incidentaloma, congenital adrenal hyperplasia, enzyme, surgery, gene, CYP21A2, myelolipomas, adrenocortical carcinoma

## Abstract

Our aim is to update the topic of adrenal tumours (ATs) in congenital adrenal hyperplasia (CAH) based on a multidisciplinary, clinical perspective via an endocrine approach. This narrative review is based on a PubMed search of full-length, English articles between January 2014 and July 2023. We included 52 original papers: 9 studies, 8 case series, and 35 single case reports. Firstly, we introduce a case-based analysis of 59 CAH-ATs cases with four types of enzymatic defects (CYP21A2, CYP17A1, CYP17B1, and HSD3B2). Secondarily, we analysed prevalence studies; their sample size varied from 53 to 26,000 individuals. AT prevalence among CAH was of 13.3–20%. CAH prevalence among individuals with previous imaging diagnosis of AT was of 0.3–3.6%. Overall, this 10-year, sample-based analysis represents one of the most complex studies in the area of CAH-ATs so far. These masses should be taken into consideration. They may reach impressive sizes of up to 30–40 cm, with compressive effects. Adrenalectomy was chosen based on an individual multidisciplinary decision. Many tumours are detected in subjects with a poor disease control, or they represent the first step toward CAH identification. We noted a left lateralization with a less clear pathogenic explanation. The most frequent tumour remains myelolipoma. The risk of adrenocortical carcinoma should not be overlooked. Noting the increasing prevalence of adrenal incidentalomas, CAH testing might be indicated to identify non-classical forms of CAH.

## 1. Introduction

Congenital adrenal hyperplasia (CAH), a cluster of diseases caused by deficiencies in the enzymes involved in steroidogenesis, impairs the synthesis of the cortisol from cholesterol [1,2,3]. The most common deficit is 21-hydroxylase (CYP21A2); it is caused by pathogenic variants in the *CYP21A2* gene, located in the long arm of chromosome 6, representing 95% of all CAH cases [4,5,6,7,8]. Other enzymatic deficits include 17 alpha-hydroxylase/17,20-lyase (CYP17A1), 11 beta-hydroxylase (CYP11B1), and more rarely, 3-beta-hydroxysteroid dehydrogenase type II (HSD3B2) [9,10,11]. The degree of CAH severity is inversely correlated to the level of residual enzymatic activity [12,13,14]. CAH diagnosis is based on the clinical presentation, hormonal panel, Adrenocorticotropic Hormone (ACTH) stimulation test, and genetic testing [15,16].

Many countries have established CAH screening programs, and thus the early identification of the condition in most classic forms is at its birth [17,18]. Prenatal diagnosis is also possible, underlying invasive methods such as analysis of the foetal hormones from amniotic fluid, chorionic villus sampling, and non-invasive procedures (cell-free foetal DNA from maternal blood) [1,19,20]. Clinical presentation varies from classical CAH, either salt-wasting (SW-CAH), representing the most severe form, or simple virilizing (SV-CAH), to non-classical CAH (NC-CAH) [1,21]. In countries without a valid neonatal screening protocol, SW-CAH is typically diagnosed at birth due to salt-wasting crises (manifested with vomiting, diarrhoea, severe dehydration, arrhythmias, and extremely low blood sodium levels). SV-CAH may be recognised later in life despite the early virilisation of the genital organs; a mild adrenal insufficiency may be associated, but there is no severe infantile hyponatremia. NC-CAH is often overlooked in daily practice; affected individuals usually present mild androgen excess symptoms [14,22,23,24,25,26].

CAH may be associated with various adrenal tumours/masses, apart from the general imaging aspects of “adrenal hyperplasia” that sometimes mimic a nodular lesion (tumour-like presentation), and the distinction is mainly established upon post-adrenalectomy histological exam. Adrenal tumours have a prevalence of up to 30% according to some authors (myelolipomas being the most frequent type); common pathogenic traits between these masses and CAH are still a matter of debate [1,27,28].

Myelolipomas, also known as myeloid lipomas, are benign, small-growing tumours that have a fat tissue component and elements of myeloid cells. Typical imaging characteristics are related to the presence of fat. Their density varies with the fat-to-myeloid component ratio, while enhancement is low due to the poor blood flow [29,30,31,32]. Sometimes they reach gigantic sizes, causing compression of nearby organs and local symptoms/signs such as abdominal pain, palpable mass, or urinary tract obstruction. Surgical treatment is electively performed in large and symptomatic tumours (those larger than 10 cm should undergo the standard approach, which is open surgery, rather than laparoscopic removal). Adrenalectomy may induce iatrogenic adrenal insufficiency, and thus a careful decision should be made [33,34,35,36,37,38]. Surgery is not usually necessary in asymptomatic myelolipomas and a normal endocrine picture [39,40].

Overall, CAH remains a challenging condition in terms of best management, adequate hormonal substitution (to avoid both over and under treatment), and most practical surveillance protocol [41,42,43]. Adrenal tumours in these patients are generally associated with a late CAH diagnosis and a poor disease control [28]. Current guidelines do not recommend the use of ACTH assays during usual CAH monitoring [1,13]. However, high persistent levels of plasma ACTH represent a potential pathogenic element of the adrenal tumours in CAH [27,28].

Moreover, in addition to myelolipomas, two other specifications should be made when it comes to the overall picture of adrenal tumours in CAH. Adrenocortical carcinoma is extremely rarely reported (and generally, it has a low prevalence in the general population, being considered an orphan disease); it is not yet clear whether the prevalence is higher in CAH than seen in the general population [1]. Correct identification is mandatory, being based on a complex panel of assessments (including imaging findings, urine steroid metabolomics, and post-operatory histological and immunohistochemistry reports) [44,45].

The increasing use of different imaging techniques of diagnosis has identified a larger number of adrenal incidentalomas [46]. It is still a matter of debate if their true frequency is higher in the CAH population or if these patients are the subject of an increased number of imaging evaluations (thus a false increase of their incidence involves individuals with CAH) [46]. Even though NC-CAH poses lower risks compared with SW-CAH and SV-CAH, considering the need for adequate fertility management, genetic advice, and even prenatal treatment in NC-CAH, it is crucial to correctly identify these masses and a careful strategy of intervention or surveillance should be taken into consideration, mostly based on an individual decision according to a multidisciplinary team [47,48,49]. The underlying pathological exam in CAH-related incidentalomas includes myelolipomas, adrenocortical adenomas (and even carcinomas), or typical/atypical hyperplasia [46,47,48,49].

Adrenalectomy is not routinely indicated due to the associated risks of the surgery itself and potential endocrine issues of developing adrenal insufficiency. The question of whether the benefits of adrenalectomy surpass the risks in patients with myelolipomas remains a current matter of interest, while generally incidentalomas need a complete hormonal workup and close surveillance in CAH subjects [46,47,48,49].

### Aim

Our purpose is to update adrenal tumour profiles in patients diagnosed with CAH. This is a multidisciplinary, clinical perspective based on an endocrine approach.

## 2. Material and Methods

This is a narrative review of the English-published, full-length papers on the topic of CAH and adrenal tumours (between January 2014 and July 2023). We performed a PubMed-based search with the following keywords: “21 hydroxylase deficiency”, “11 beta hydroxylase deficiency”, “17 alpha hydroxylase deficiency”, “3 beta hydroxysteroid dehydrogenase deficiency”, “17 beta hydroxysteroid dehydrogenase” in different associations with “tumour”, “myelolipoma”, “adrenocortical carcinoma”, “adrenal carcinoma”, “neuroblastoma”, “adrenal tumour”, “adrenal adenoma”, and “incidentaloma”.

The search strategy was dual: identifying adrenal tumours identified in CAH patients or CAH as the underlying diagnosis in patients already confirmed with different adrenal masses. We only included the papers in which the term adrenal „tumour” or „mass” was specified by the original authors and not the studies that introduced the usual adrenal hyperplasia imaging aspects in CAH. We included original studies in humans and excluded experimental, in vitro, and animal studies.

Notably, we kept the original terms “myelolipoma”, “adrenocortical carcinoma”, and “incidentaloma” that were introduced by the cited papers according the original authors, despite the fact that the first two can behave as an incidentaloma as well, but then the tumour should be further characterised based on imaging investigations, hormonal work-ups, histological reports (if available), etc. Statistical analysis was based on prior published data (original articles); we used mean, median, standard deviation, and ranges depending on the parameter. As mentioned, the included original articles were not restricted by the level of statistical significance.

Moreover, we mention that the cases diagnosed with CAH were included regardless of the fact that CAH diagnosis was established only based on the endocrine workup, but not genetically confirmed by the original authors (and we specified this aspect), since real life medicine showed us that in many centres genetic testing is not unanimously available, yet the diagnosis of CAH can be clearly established upon clinical and hormonal assessment [41,42,43]. Of very important note, the adrenal tumours described in the patients diagnosed with CAH were not limited to the presence of the pathological report following adrenalectomy if the original authors specifically provided the imaging features of the tumour (in the absence of adrenal surgery). This is because we did not intend to restrict the search to the histological profile after surgery and since not all the clinicians agree that each tumour detected in one patient with CAH should be removed [1,27,28].

## 3. Results

After applying the mentioned strategy, we finally included a total of 52 original works: 9 studies, 35 single case reports, and 8 case series. We organised this sample-based, 10-year study according to two main sections: one is represented by a case-based analysis (3.1.) enrolling reports of CAH and adrenal tumours according to our strategy of search. We followed the genetic/enzymatic defects (CYP21A2 in Section 3.1.1., CYP17A1 in Section 3.1.2., CYP17B1 in Section 3.1.3, and HSD3B2 deficiency in Section 3.1.4.) and analysed the clinical presentation, genetic testing results, hormonal work-up, as well as adrenal mass features such as size, histological report (if available), management, and outcome at the moment when these tumours were confirmed in CAH patients.

**Figure 1:**
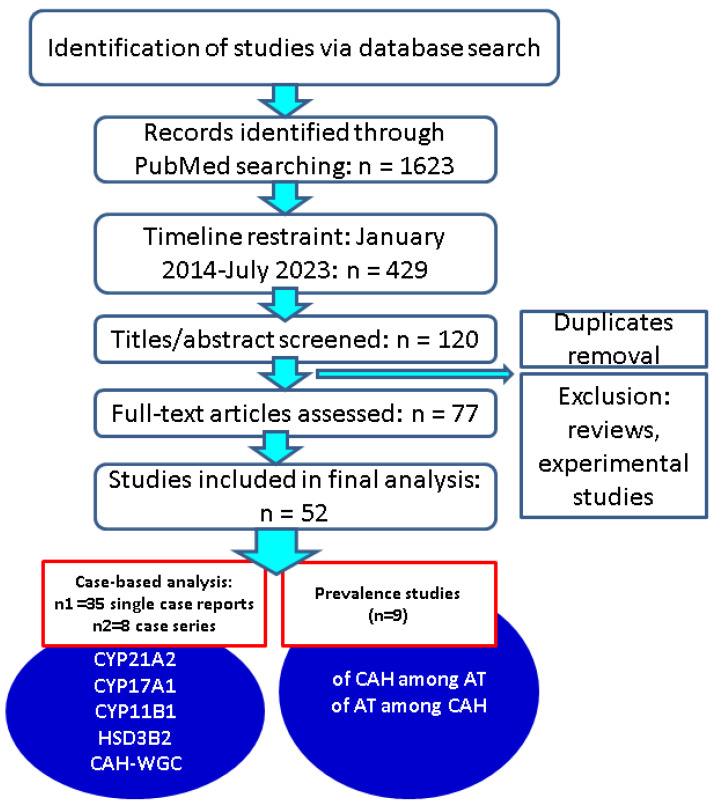
Strategy of research—associated flowchart (n = number of articles). Approach of CAH—adrenal tumours connections. Abbreviations: CAH = congenital adrenal hyperplasia; n = number of studies; AT = adrenal tumours; WGC = without a genetic confirmation; CYP21A2, CYP17A1, CYP11B1, HSD3B2 = enzymes/genes deficiencies; n = number of studies.

The second section, Section 3.2, includes larger original studies (not case reports or series) where the prevalence of the adrenal tumours in CAH or of CAH in adrenal tumours was reported amid different clinical, genetic, or imaging characteristics (prevalence studies) (Figure 1).

### 3.1. Case-Based Analysis of Synchronous CAH and Adrenal Tumours

#### 3.1.1. CYP21A2 Deficiency

##### 3.1.1.1. Characteristics of the Patients

A total of 27 patients [50,51,52,53,54,55,56,57,58,59,60,61,62,63,64,65,66,67], 19 phenotypically males [53,54,55,56,57,58,60,61,62,63,64,65,66,67] and 7 phenotypically females [50,51,58], were identified according to our strategy (n = 18 case reports and series). Two male subjects had 46,XX karyotype [57,62]. Overall (N = 27), the median age at presentation was 47 years. The youngest patient was 21 [64], while the oldest was 88 [51]. The median age at CAH diagnosis was 45.5. The median age at tumour diagnosis in these CAH individuals was 46. Most of these mentioned patients with pathogenic variants of *CYP21A2* were diagnosed with CAH after the discovery of an adrenal tumour (N = 20) [51,52,53,54,55,56,57,58,59,60,61,62,63,64,66,67]. Only five persons were previously known with CAH [50,54,64,65] (two more cases’ data were not available). In terms of CAH form, most patients presented with SV-CAH (N = 13) [50,51,55,56,57,58,61,62,63,64,66], followed by NC-CAH (N = 11) [51,52,53,54,59,60,66,67]; only two patients had SW-CAH [64,65], while another subject had a carrier status [51] (Table 1).

##### 3.1.1.2. Features of the Adrenal Tumours/Masses in Patients with CYP21A2 Deficiency

Most tumours were myelolipomas (N = 12/27 patients) [50,54,56,58,60,61,62,63,65,67], followed by incidentalomas (N = 10) [51,53,55,59,64,66]. Two adrenal incidentalomas were classified as adrenocortical adenoma following the post-adrenalectomy pathological report [59,64]; two adrenal masses were classified as hyperplasia, one being an adenomatous hyperplasia [67] and the other “hyperplasia with mild atypia” according to the original terms [57]. Adrenocortical carcinoma was also identified in one patient (1/26) [52]. One subject had both a myelolipoma and an ectopic (renal) adrenocortical adenoma [63], and another suffered from two bilateral myelolipomas and ectopic adrenal rests [65].

The majority of the mentioned cases were unilateral (N = 14, representing 51.8% of the entire cohort). The left adrenal gland [51,52,53,55,59,64] was affected more frequently (63.28% of the unilateral tumours, N = 9) and more severely compared with the right adrenal gland [45,51,57,67]. Bilateral tumours were found in 48.14% of the persons (N = 13); in seven cases, the largest tumour was located in the left adrenal [50,51,54,56,61,66].

A total of 10/27 patients had tumours smaller than 5 cm [51,52,59,63,64,67], while nine tumours were between 5 and 10 cm [51,53,55,57,58,63,65,66], respectively, and the other masses (N = 7) were larger than 10 cm [50,54,56,61,65,67]. Two other tumours were described as “gigantic”, but data regarding the exact size were not provided [60]. Of note, the largest tumours in subjects with genetically confirmed *CYP21A2* deficiency were bilateral adrenal masses of 30 by 40 cm on the right, respectively, and of 20 by 25 cm on the left [67]. Increased tumour size caused compression of the nearby organs. Mallappa et al. [58] presented a case of CAH recognition after adrenal tumour diagnosis starting from hyperpigmentation, short stature, and signs of virilisation upon admission for abdominal pain due to kidney compression. A 17-hydroxyprogesterone level 100 times the upper normal limit was confirmed as well as *CYP21A2* gene heterozygosity for intron 2 IVS2-13A/C>G splice site pathogenic variant/p.R483P (c.1451_1452 deletion insertion of C) [59] (Table 2).

##### 3.1.1.3. Management and Outcome of the Adrenal Tumours/Masses in Patients with CYP21A2 Deficiency

Most tumours were surgically removed (N = 16) [50,51,52,54,57,58,59,60,61,62,63,64,65,66,67]; glucocorticoid replacement was offered in 29.6% of the cases (N = 8) [51,53,55,56,59,62]; lifestyle intervention was recommended in one subject (4%) [51]; another refused any medical intervention [51]. Six out of seven patients with tumours over 10 cm underwent surgery (one patient underwent unilateral adrenalectomy two times) [50,54,61,65,67]; yet, another subject with large bilateral myelolipomas of 14 cm at the largest diameter received glucocorticoid treatment; he died shortly after diagnosis due to prostate cancer metastases [56]. Adrenalectomy was performed as well in three patients with tumours <5 cm [52,64], one having an adrenocortical carcinoma of 2 cm [52].

Long-term outcomes are poorly reflected by the published data. Following adrenalectomy, an event-free course at 6 and 12 months follow-up were mentioned in one case [54], respectively, and another subject was offered hydrocortisone and the adrenal mass remained stationary during long-term surveillance [53]. Treatment with glucocorticoids such as dexamethasone was associated with a favourable outcome in another subject with tumour decrease. This is the report of Buitenwerf et al. [55] introducing a 43-year-old male with a left adrenal incidentaloma of 5.2 by 4.4 cm that was accidentally discovered on a computed tomography scan. CAH diagnosis was based on biochemical evaluation, including the response to the ACTH stimulation test. Genetic testing confirmed compound heterozygous genetic variants: c.518T>A (p.Ile173Asn) and c.710T>A, c.713T>A, c.719T>A (p.lIe237Asn), (p.Val238Glu), (p.Met240Lys). After dexamethasone administration (0.5 mg/day) for 1 year, the tumour decreased to 4.4 by 2.9 cm [55].

The most frequent presentation was with abdominal symptoms, namely abdominal pain (N = 5) [50,51,58,60], abdominal discomfort (N = 3) [51] or distention (N = 1) [67], and palpable mass (>10 cm) [54]; alternatively, back pain was reported [63]. Two CAH subjects were admitted for adrenal insufficiency [59,62], one of them being diagnosed 22 years following unilateral adrenalectomy [59]; the case of adrenocortical carcinoma was admitted for gynecomastia [52].

Paradoxically, a 61-year-old male was diagnosed with CAH after the discovery of bilateral myelolipomas (of 18.2 and 7.8 cm maximum diameter, respectively) in association with persistent increased testosterone despite the fact that he was under antiandrogen treatment (leuprolide and bicalutamide) for prostate cancer. The patient received dexamethasone therapy due to lethargy and fatigue, with prompt improvement of symptoms and a decrease in testosterone levels. Genetic testing revealed a CYP21A2 deficiency through bi-allelic genetic variants in the *CYP21A2* gene: complete gene deletion on one allele and a C518T>A (I172N) genetic variant on the other [56].

Urinary tract symptoms represent an alternative to the clinical (abdominal) features on first admission. For example, Hui et al. [57] introduced a 65-year-old individual with late CAH diagnosis; the phenotypically male presented lower urinary tract symptoms. Further on, the clinical examination revealed an empty scrotum and small penile length as well as short stature. Increased 17-hydroxyprogesteron and estradiol levels as well as the urinary profile of the steroid metabolites suggested a CYP21A2 deficiency, confirmed by genetic testing (compound heterozygous: p.Ile172Asn, p.Arg483Pro, and p.Met485Trpfs*56 genetic variants); chromosome testing confirmed a 46,XX karyotype. The patient underwent right adrenalectomy due to the discovery of a right adrenal mass of 5.8 cm maximum diameter on computed tomography imaging (that was initially performed in search of intra-abdominal gonads). Pathological examination confirmed adrenal hyperplasia with a mild atypia [57] (Table 3).

##### 3.1.1.4. The Analysis of CAH (Disease) Control in Relationship with Tumour Status

We identified 11 reports that provided data regarding ACTH values for 40% (N = 11) patients [50,53,54,55,59,61,62,63,65,66,67]. ACTH was increased in 9/11 subjects, out of which 3 had tumours >10 cm [50,54,56,61]; 3 individuals had adrenal tumours between 5 and 10 cm [55,62,66], while another 3 persons had tumours <5 cm [59,63,67]. In these patients, ACTH values ranged between 27 pg/mL [55] and 10,445 pg/mL [62], with an average of 1830 pg/mL and a median of 1131 pg/mL. The mean ACTH levels in relationship with the tumour size were 514.5 pg/mL (tumours >10 cm), 3544 pg/mL (tumours: 5–10 cm), and 1434 pg/mL (tumours <5 cm). Of note, the highest ACTH occurred in a subject with bilateral myelolipomas of 6 cm maximum diameter [62], and the lowest ACTH was assessed in a patient with a left adrenal tumour of 5.2 cm [55]. Moreover, normal ACTH was confirmed on an individual with a left adrenal tumour of 5.5 cm by 3.6 cm by 4.5 cm [53].

In terms of 17-hydroxyprogesteron values, 14/18 studies provided data regarding this hormone, out of which 5 had tumours >10 cm [50,54,61,65], 6 had tumours of 5–10 cm [51,53,55,58,62,66], and 7 had tumours <5 cm [51,52,59,63,67]. The levels ranged between 940 ng/dL and 27,500 ng/dL (average of 8499 ng/dL; median of 5700 ng/dL). The mean value, depending on tumour size, was 15,390 ng/dL (tumours >10 cm), 8818.8 ng/dL (tumours of 5–10 cm), and 4287.6 ng/dL (tumours <5 cm); the highest value was identified in an individual with a left adrenal mass of 12.5 cm maximum size in association with a diffuse nodular enlargement of the right adrenal [61], while the lowest was diagnosed in a patient with a left adrenal mass of 3–4 cm [59].

Late CAH diagnosis was present in at least 12 patients [51,52,56,57,58,59,60,61,62,63,66,67], while lack of treatment or poor compliance with CAH therapy was specifically addressed in some cases [50,59,64]. Adequate diagnosis in CAH might bring unexpected results, such as the identification of ectopic myelolipomas [63]. Two other cases of myelolipoma and uncontrolled CAH were reported by Almeida et al. [50]. Another sign of poor disease control at the moment of adrenal tumour co-diagnosis was hyperpigmentation (N = 3) [58,59,62] (Table 4).

#### 3.1.2. CYP17A1 Deficiency

A total of six patients, all phenotypically female, but one with a 46,XY karyotype 7, were identified with CYP17A1 deficiency across three single case reports and one case series of five patients with myelolipomas (out of fifteen individuals tested for CAH genetic variants belonging to two unrelated families), and three of them were identified with the mentioned enzymatic defect [60,68,69,70].

The average age at presentation was 33.1 years, with a median of 34. The youngest subject was 27 years old [69], while the oldest was 37 [60]. Five out of six individuals presented myelolipomas [60,69,70]; one subject out of six was confirmed with an adrenocortical adenoma [68]; 50% (N = three) of the sample-based cohort had bilateral tumours [60,69]. All unilateral tumours (N = three subjects) were on the left adrenal gland [60,68,70]. The largest adrenal tumour was found by Liu et al. [60] (20 by 15 by 10 cm). This patient had giant bilateral tumours and shared a compound heterozygous genetic variant (c.1118A>T, p.H373L, c.1459_1467del9, and p.D487_F489del) with two of her sisters [60]. The dimensions of the adrenal mass in an individual harbouring a tumour are 10 by 6.3 by 8.6 cm, associated with a compound heterozygous genetic variant for p.Tyr329fs (c.985_987delTACinsAA) and a missense genetic variant p.His373Leu (c.1118A>T) [68]. Moreover, Chang et al. [70] reported a left adrenal mass of 5 by 9 cm associated with a genetic profile of a heterozygous variant of c.985_987delinsAA (p.Y329Kfs*90) and the p.R96W genetic variant [70].

Five of six patients with CYP17A1 deficiency had hypokalaemia at the moment when the adrenal tumour was confirmed [60,69,70]. Arterial hypertension was also noted in three cases (50%) [60,70]; other symptoms included headaches and fatigue [60]; only one individual had abdominal pain due to a tumour of >10 cm [68].

All subjects (N = 6) had high ACTH or high normal ACTH, with values ranging from 41.56 pg/mL [69] and 1250 pg/mL [60], with an average of 394.5 pg/mL; the highest ACTH value was found in the patient with the largest tumour [60]. However, an ACTH—tumour size correlation was not confirmed due to a small sample size. Plasma cortisol levels were provided in five of six persons, all of whom had low values [60,68,69,70]. Aldosterone was increased in two cases (if available) [68,70]. Data regarding 17-hydroxyprogesterone were provided for one female who presented with a very low level of < 0.05 ng/mL [69].

Five of six subjects underwent adrenal surgery [60,68,70]; one patient out of six was managed with dexamethasone 0.75 mg per day and showed a reduction of the adrenal tumour diameter [69].

In terms of outcome, five of six patients had an adrenal tumour removal [60,68,70]. The patient of Lee et al. [68] postoperatively developed nausea, weakness in association with elevated ACTH, and blunted cortisol response to stimulation test. At the 3-year follow-up, progression of the right adrenal hyperplasia was associated with high ACTH levels and poor compliance with glucocorticoid treatment [68] (Table 5).

#### 3.1.3. CYP11B1 Deficiency

We identified one case report with a CYP11B1 deficiency. Ozbas et al. [71] presented a 35-year-old female with genital reconstruction during childhood who suffered from hypertension science at the age of 12. She had a left adrenal mass of 7.4 cm associated with hypokalaemia, high ACTH, and androstenedione. Adrenalectomy had a favourable outcome, with post-surgery confirmation of a myelolipoma. Genetic analysis revealed a homozygous missense genetic variant: c.1385T>C L462P variant (NM_000497.3) in the CYP11B1 gene [71] (Table 6).

#### 3.1.4. HSD3B2 Deficiency

We mention an analysis coming from a larger study on HSD3B2 deficiency. Ladjouze et al. [72] studied 14 out of 273 patients with classic CAH who suffered from HSD3B2 deficiency. Out of these 14 individuals (coming from 10 families), 3 females had adrenal tumours and SW-CAH (all with a history of consanguinity). CAH diagnosis was established early (13, 15, and 16 years, respectively; two of them were sisters). Both siblings carried the p.(Thr152_Pro155del) genetic variant. One of them had a large left adrenal mass of 6.3 by 5.2 by 5.1 cm. Histological examination following adrenalectomy proved an adrenocortical hyperplasia in one case, while her sister presented a smaller left adrenal mass of 2.5 cm and she continued surveillance. The third patient, harbouring a p.(Pro222Gln) pathogenic variant, had a right adrenal mass (of 3 cm) with necrotic areas that was removed due to a high malignancy suspicion; however, the histological report also showed an adrenal hyperplasia. This patient was associated with an ovarian adrenal rest tumour [72].

Overall, the topic of the adrenal tumours/masses in cases with CYP11B1 and HSD3B2 defects remains at a low level of statistical evidence and awareness is necessary, while no clear hormonal—adrenal tumour correlations can be established so far (Table 7).

#### 3.1.5. Adrenal Tumours in Patients Diagnosed with CAH without a Genetic Confirmation

We identified 18 case reports and 1 case series (N = 22 patients) of CAH and adrenal tumours where genetic testing was either not performed (N = 17) or negative (N = 5). Most subjects were diagnosed with CYP21A2 deficiency (N = 17) [64,73,74,75,76,77,78,79,80,81,82,83,84,85,86,87,88,89], while another had CYP17A1 deficiency [89]; four other cases did not have a specific enzymatic deficit available (according to a limited genetic testing panel), but considering that CYP21A2 has the highest prevalence, they would be very likely to suffer from it [85,86,87,88].

The scenario of detecting an adrenal tumour starts from an adrenal imaging scan; an individual decision is taken with respect to adrenalectomy. For example, we mention a 26-year-old patient who presented with hypertension and hypokalaemia who was diagnosed with 17α-hydroxylase/17,20-lyase deficiency according to biochemical and endocrine findings (low plasma cortisol, 17-hydroxyprogesteron, respectively, high ACTH and deoxycorticosterone, as well as hypogonadotropic hypogonadism). Genetic testing for CYP17A1 genetic variants was unavailable. The patient’s karyotype was 46,XY. Magnetic resonance imaging did not reveal any gonads. Glucocorticoids and oestrogens were initiated. A left adrenalectomy was performed due to an asymmetrical enlargement of the left adrenal and associated persistent abdominal pain. A pathological examination confirmed a myelolipoma [89].

Out of the 22 patients, 15 were phenotypically male [51,73,75,76,77,78,79,80,81,82,83,85,87,88] and 4 were phenotypically female [51,74,84]; one phenotypically male had 46,XX karyotype [82]; one phenotypically female had 46,XY karyotype [89]; and one subject born female was identifying as male [86]. The mean age at adrenal tumour identification was 44.5 years (a median of 43). The youngest patient was 26 years old [89], and the oldest was 68 years old [84] (Table 8).

Most patients were diagnosed with CAH before the actual discovery of an adrenal tumour (N = 13/22); some of them displayed an early CAH confirmation. For example, three were diagnosed as neonates [78,86,87], and seven of them were diagnosed during infancy or childhood [64,73,74,75,76,82,83,88]; two subjects had an uncertain time of CAH diagnosis before presentation for adrenal tumours [81,85]). On the opposite side, 9/22 individuals were identified with CAH after the discovery of the adrenal masses [51,77,79,80,84,89]. Late CAH confirmation was established in some individuals (N = 5) [77,79,80,84,89], while others, despite prior diagnosis, were poorly compliant with specific CAH therapy (N = 5) [73,74,86,87,88].

In terms of CAH form, most patients presented with SV-CAH (N = 7) [64,74,76,79,80,81,82], followed by SW-CAH (N = 6) [75,78,83,86,87,88] and NC-CAH (N = 5) [51,84]; there was also one carrier [51]. The most frequent tumours were myelolipomas (N = 14) [64,73,74,75,76,79,80,81,84,85,86,87,88,89], respectively, incidentalomas (N = 8) [51,79,80,82,84], adrenocortical adenoma (N = 2) [77,82], and adrenocortical carcinoma (N = 2) [78,81] and lipoma (N = 1) [83].

The majority of the tumours were bilateral (N = 18/22 subjects, 13 of them having a larger mass on the left than right side) [51,64,73,75,76,78,84,86,87,88,89]; 4/22 had unilateral adrenal masses (all of them being located at the left adrenal gland) [74,80,82,85]. Most tumours were >10 cm (N = 12) [73,74,75,76,78,81,83,84,85,86,87,88], and 5/12 were larger than 20 cm [73,74,75,85,87], the largest being >30 cm [75,85]; five subjects had tumours of 5–10 cm [77,79,80,82,89], and four persons had tumours <5 cm [51,64]. Of note, all patients with SW-CAH had adrenal masses >10 cm [75,78,83,86,87,88]. The largest myelolipoma was reported by Longoria-Dubocq et al. [85] in a 36-year-old male with prior known CA associated with good adherence to the treatment and regular follow-up. He presented abdominal pain; a computed tomography scan found a left retroperitoneal mass of 30 by 23.6 by 16.7 cm, being referred to adrenalectomy (myelolipoma) [85]. Kale et al. [75] reported the second largest myelolipoma on a 51-year-old male with SW-CAH identified during infancy [75]. Overall, most subjects who were confirmed to have unilateral or bilateral adrenal tumours underwent adrenalectomy (N = 15/22) [73,74,75,76,77,78,79,81,82,83,84,85,86,87,88,89].

The clinical picture at the moment of the adrenal tumour recognition mostly involved abdominal complaints such as pain [52,76,85], distension [73,74,86], nausea, and vomiting [74,76,86]. Other elements included back pain [75], lower limbs paresthesiasis [75], dysuria [83], and one case of acute adrenal insufficiency (including hyperpigmentation) [77], but there was also one case with CYP17A1 deficiency-related arterial hypertension [89].

Data regarding the outcomes in these individuals (N = 22) are relatively scarce, varying from a good evolution with symptoms remission [76,83], disease-free following adrenalectomy [82] to rapid relapse (one patient had a nodularity at the adrenal bed at 3-month follow-up) [88].

Hormonal panels were focused on CAH diagnosis depending on the specific enzyme defect. Eleven studies provided data regarding ACTH values for 11/22 subjects [73,77,79,80,81,82,83,84,85,86,87,88,89,90,91,92,93,94,95,96,97]; the hormone was increased in 8/11 cases [77,79,80,81,84,86,87,89], out of which 4/8 had tumours >10 cm [81,84,86,87], and 4/8 had tumours of 5–10 cm [77,79,80,89]; ranges varied between 37.5 pg/mL (which is a normal value; overall, N = 3 individuals had normal ACTH) [83] and 2000 pg/mL [77] (average of 342.9 pg/mL; median of 166 pg/mL). Tumour size—ACTH analyses showed in cases with masses larger than 10 cm, respectively, between 5 and 10 cm, a mean ACTH of 128 pg/mL and 753.8 pg/mL, respectively. Interestingly, the highest ACTH was registered in one patient with a right adrenal mass of 5 cm and a left adrenal mass of 4.1 cm that were found 9 years after a right adrenalectomy was performed [77], while the lowest value was confirmed in a subject with bilateral adrenal masses of 10 cm and 19 cm, respectively [83]. Overall, 3/22 tumours were confirmed at the moment of good disease (CAH) control, and that is why a tide association is difficult to establish [82,83,85].

In terms of 17-hydroxyprogesterone values, 13 studies provided these values [51,73,77,79,80,81,82,83,84,86,87,88,89], namely including a total of 16/22 patients (7/16 had adrenal masses larger than >10 cm). The values varied between 10 and 25,018 ng/dL (a median of 1756 ng/dL); hormone value—tumour size analysis showed a mean level of 11,521 ng/dL (tumours >10 cm), 4330 ng/dL (tumours of 5–10 cm), and 219.5 ng/dL (tumours <5 cm). Of note, the highest value occurred in a patient with bilateral adrenal masses of 10.5 cm (left) and 7.6 cm (right) [84] (Table 9).

### 3.2. Prevalence Studies on CAH and Adrenal Tumours

Apart from the mentioned case reports/series, nine studies [90,91,92,93,94,95,96,97,98] addressed the issue of CAH and adrenal tumours from different perspectives, such as CAH prevalence among the individuals diagnosed with adrenal tumours/incidentalomas [90,91,92] or long-term outcomes in relationship with disease control and adrenal morphology, particularly, the identification of distinct adrenal tumours [93,94,95,96,97,98].

A study investigating the relationship between CAH control and adrenal imaging aspects was conducted by Kim et al. [96]; the retrospective cohort included 90 adults with 21-hydroxlase deficiency and 270 healthy controls; 12/90 (13.3%) subjects were diagnosed with adrenal tumours: unilateral (N = 9/12) and bilateral (N = 3/12). Except for one adrenocortical adenoma, all tumours were identified as myelolipomas based on radiologic (computed tomography) findings. Higher levels of ACTH, 11β-hydroxyandrostenedione, and progesterone sulphate levels were associated with the presence of these masses, but no correlation between hormonal values and tumour size was established [96]. Similarly, a retrospective study on 88 patients with classic CAH revealed a myelolipoma prevalence of 12.5% (N = 11), respectively, of benign adenomas of 9% (N = 8); one pheochromocytoma was reported as well (this represents the only case reported during the latest 10 years) [95]. Another study regarding long-term consequences of CAH (CYP21A2 deficiency) followed 20 subjects (among a total of 53) via adrenal ultrasound: 3/20 subjects with SV-CAH had adrenal adenomas; 1/20 cases of SV-CAH was associated with adrenocortical carcinoma at the age of 15; 1/20 patients with SV-CAH presented a myelolipoma [93].

The prevalence of CAH in subjects diagnosed with adrenal incidentalomas varied between 0.3% and 3.6% [90,91,92,94,97,98,99]. Kiedrowickz et al. [91] analysed the prevalence of NC-CAH among adults with adrenal incidentalomas (N = 100). The most common genetic variants of CYP21A2 were tested; 8/100 (0.8%) persons had CYP21A2 pathogenic variants (and 3/8 of them exceeded the cut-offs for stimulated 17-hydroxyprogesterone). When compared to a control group of healthy neonates (as a substitute for the prevalence of these genetic variants in the general population), the *CYP21A2* genetic variant prevalence was statistically significantly higher in patients confirmed to have incidentalomas (N = 3 subjects with P30L, N = 3 with P453S, and N = 2 with V281L). The mean tumour size was 2.4 cm (between 1.6 cm and 3.7 cm) [91]. Similarly, Patrova et al. [92] analysed 637 patients with adrenal incidentalomas from the perspective of underlying CAH; 2/637 (0.3%) were confirmed to have CAH; both cases have been previously reported [51,100]. A bias of interpretation of this rate came from the fact that not all the patients were specifically tested for CAH. Yet, a subgroup was assessed either by urinary steroids profile (N = 26) or by 17-hydroxyprogesterone assays (N = 47), and thus CAH prevalence increased to 2.9% [92]. A prospective cohort of 228 adrenal incidentalomas identified late-onset CAH in 1.8% of them (N = 4) [94]. Sahlander et al. [97] analysed CAH prevalence in 320 subjects with adrenal incidentalomas by using an ACTH stimulation test and genetic confirmation; 8/222, representing 3.6%, had NC-CAH (only this subgroup of 222 individuals was assessed via the mentioned dynamic test; a cut-off of 17-hydroxyprogesterone of ≥30 nmol/L was applied) based on an ACTH stimulation test and not basal levels (with negative genetic testing with respect to *CYP21A2* gene). Fifty percent of the individuals with NC-CAH based on an ACTH stimulation test had bilateral adrenal tumours (and the others had unilateral masses). A larger size was correlated with a positive stimulation test, with diameters ranging from 1.6 to 6.6 cm (median of 3.8 cm) [97].

A retrospective, register-based study on 26,573 individuals with adrenal tumours versus 144,124 controls (without adrenal tumours) analysed the prevalence of CAH, which was 0.75‰ (N = 20) versus 0.0007‰ (N = 1); there was an odds ratio (OR) of 109, and 95% CI: 15–809 (*p* < 0.0001). Regarding the CAH subgroup with adrenal masses (N1 + N2), the timeline relationship was as follows: 5 subjects had a CAH diagnosis before the tumour identification (N1 = 5) and 15 individuals were confirmed to have CAH after the tumour finding (N2 = 15). The mean age at tumour diagnosis was 55.6 ± 14.7 years (N1 + N2); N1 seemed to be older than N2 (of 58 ± 14 *versus* 47.6 ± 15.7 years), but the difference was not statistically significant. However, the perspective of analysing the age at CAH diagnosis showed a statistically significant decrease of N1 versus N2 (11.8 ± 26.4 versus 60.7 ± 13.5 years). N1 included SW-CAH or SV-CAH (80%) and one case of suspected NC-CAH (20%); N2 included NC-CAH (80%, N = 12) and SV-CAH (20%, N = 3). Adrenalectomy was performed in 35% (N = 7) of the subgroups N1 + N2 (five unilateral and two bilateral adrenalectomy coming from N1 subgroup). All resected tumours were non-functioning, benign adenomas of the cortex (adrenocortical carcinoma was suspected, but not confirmed, in two cases). Of note, two patients died of an adrenal crisis 2 years and 9 years, respectively, following the initial tumour diagnosis [98]. Moreover, Askitis et al. [90] investigated 187 participants with adrenal tumours and identified one case (0.53%) of CAH (a 49-year-old male who was diagnosed during kidney transplant investigations) [90] (Table 10).

## 4. Discussion

### 4.1. From Case-Sample Analysis to Prevalence Studies

**Figure 2:**
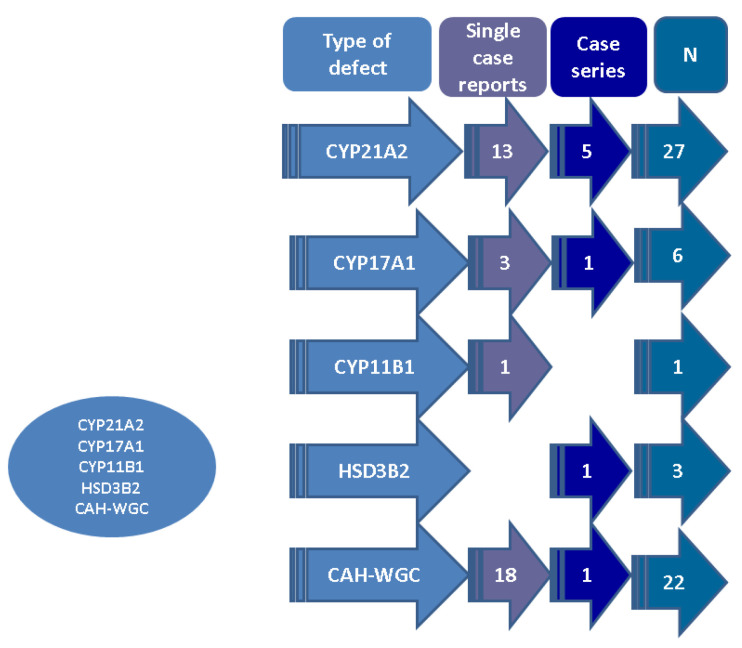
Case-sample analysis (n = 43 original papers, N = 59 patients; overall publication timeline between 2014 and 2023). Abbreviations: CAH = congenital adrenal hyperplasia; N = number of patients; WGC = without a genetic confirmation.

The analysis of published case reports and series showed, as expected, that the majority of data concerned 21-hydroxylase deficiency (13 single case reports and 5 case series, with a maximum of 5 patients per paper with respect to 27 subjects diagnosed with synchronous CAH that was genetically confirmed and adrenal tumours), followed by CYP17A1 deficiency (3 single case reports, 1 case series with 3 patients/paper, N = 6), CYP11B1 deficiency (1 single case report, N = 1), and HSD3B2 deficiency (1 case series; N = 3); respectively, there were cases of CAH without a specific genetic confirmation (18 case reports and 1 case series; N = 22), and thus there was a total of 59 patients across 43 original papers (Figure 2).

Most patients with genetically confirmed CAH were diagnosed after the discovery of the adrenal tumours [50,51,52,53,54,55,56,57,58,59,60,61,62,63,64,65,66,67,68,69,70], while the majority of the individuals without a genetic confirmation had a known (prior) diagnosis of CAH at presentation for the adrenal mass [64,73,74,75,76,78,85,86,87,88]. SV-CAH was the most prevalent form in both mentioned groups (with genetic confirmation and without). The highest rate among tumours was for myelolipoma (N = 42), and all patients with CYP17A1 deficiency and the one with CYP11B1 deficit had this type; however, two-thirds of subjects with HSD3B2 deficiency actually had adrenocortical hyperplasia. Incidentaloma (N = 18) was the second-most frequent tumour, but this radiological term might underline different histological reports (if surgery is decided), including myelolipomas [51,52,55,59,64,65,66,79,80,82,84]. Although rare, adrenocortical carcinoma was reported in three patients [52], and it has been reported before our timeframe of research [99]. The largest diameter was 40 cm (in a case of a myelolipoma) [67].

The most common presentation in both groups included abdominal symptoms such as abdominal discomfort, pain, distension, nausea, and vomiting (either caused by a large adrenal mass or by adrenal insufficiency) [57,59,62,75,77,83]. In terms of treatment, most patients underwent surgery, especially for adrenal lesions larger than 10 cm.

In both groups, ACTH values (if available) were increased in most subjects; ACTH values did not correlate with tumour size. 17-hydroxyprogesterone was elevated as well, and it seemed higher in individuals with larger tumours (with regard to both groups of genetically confirmed and unconfirmed CAH).

Considering that genetic confirmation is not always commonly available, there are still many reports of patients diagnosed with CAH based on hormonal profiles or ACTH stimulation tests. Some of them, however, were diagnosed at birth with SW-CAH forms. Current findings suggest an under diagnosis of SV-CAH, especially in patients with concurrent adrenal tumours. Lack of treatment leading to increased ACTH (sometimes clinically expressed as hyperpigmentation) [58] may induce a tumour growth up to very large diameters of over 10 cm, affecting nearby organs, including kidney compression [58]. Patients suffering from SV-CAH may benefit from an early diagnosis and treatment, including the scenario of developing an adrenal mass.

Regarding laterality, the left side seemed more affected in cases of unilateral lesions or larger on the left adrenal in cases of bilateral tumours. A bias in recognition due to anatomical differences was suggested as a potential explanation [100,101,102,103,104]. Another explanation may be related to the morphological differences, leading to a predisposition for left adrenal tumours. These include a higher volume and therefore a larger pool of cells, asymmetries of the vascular supply and drainage, as well as differences in gland innervation [101].

Moreover, as mentioned, prevalence studies showed that awareness of CAH and adrenal tumour combination is necessary, either starting from patients who received a diagnosis of an adrenal tumour (particularly, of an incidentaloma) or of CAH. Two prospective and seven retrospective (including a registry-based cohort) studies have addressed the present topic, thus providing a good level of statistical evidence, enrolling from 53/88 patients (the smallest sample sizes) to more than 26,000 individuals, depending on the study design. Among CAH cases, adrenal tumours were reported in up to 13.3% of them [96], respectively, 20% [95], while among individuals with previous imaging diagnosis of an adrenal tumour/incidentaloma, the rate of detecting CAH was 0.3% [92], 0.53% [90], 0.8% [91], 1.8% [94], 2.9% [92], 3.6% [97], and 0.75‰ [98]. An important aspect remains the lack of systematic genetic confirmation in many of these patients.

### 4.2. Histological Profile of CAH-Associated Adrenal Tumours

The most frequent tumour in CAH was myelolipoma, in accordance with prior published data [105,106]. Even though computed tomography-based aspects of myelolipomas consist of fat density, usually negative, there are cases of myelolipomas with a heterogeneous appearance resembling an adrenocortical carcinoma. Differential diagnosis is crucial in these cases [66]. Of note, non-adrenal sites of myelolipoma have been reported as well (for example, mediastinal or renal) in non-CAH patients [107,108]; also, adrenocortical adenoma with myelolipomatous metaplasia should be taken into consideration as an alternative differential diagnosis [109].

Adrenocortical carcinoma was reported in 3 out of 59 patients according to the sample-based analysis (5.2%) [52,78,81], this rate being 10 times more frequent than in the general population [44]. For instance, a reported case was associated with bilateral myelolipomas on a 32-year-old patient with SW-CAH diagnosed as a neonate, presented with rapidly growing bilateral masses and poor hormonal control. The largest lesion was on the left adrenal side, exceeding 19 cm. A left adrenalectomy was performed and a histological exam identified an adrenocortical carcinoma. Moreover, the patient had a history of testicular adrenal rest tumour. Mitotane was initiated while waiting for the right adrenalectomy (no genetic test was available) [78]. Generally, the condition is extremely aggressive, and it requires particular attention due to its highly aggressive potential, regardless of CAH co-presence [110,111]. Larger studies are necessary to establish if adrenocortical carcinoma is indeed more frequent among CAH patients or if it is a mere coincidence. According to current data and considering the severity of this disease, the possibility of an adrenal tumour in a patient with CAH being carcinoma should not be overlooked [52,78,81].

Whether hormonal panels, particularly high ACTH and 17-hydroxyprogesterone, are part of the pathogenic elements in CAH-associated tumours is yet to be established. A link between CAH control and the development of adrenal tumours was based on the observations that a high prevalence of late diagnosis and non-compliance with treatment was registered among these patients. In subjects with 3BHSD deficiency, 17-hydroxypregnenolone might be a better predictor of disease control and tumour risk than 17-hydroxyprogesterone [71,72].

**Figure 3:**
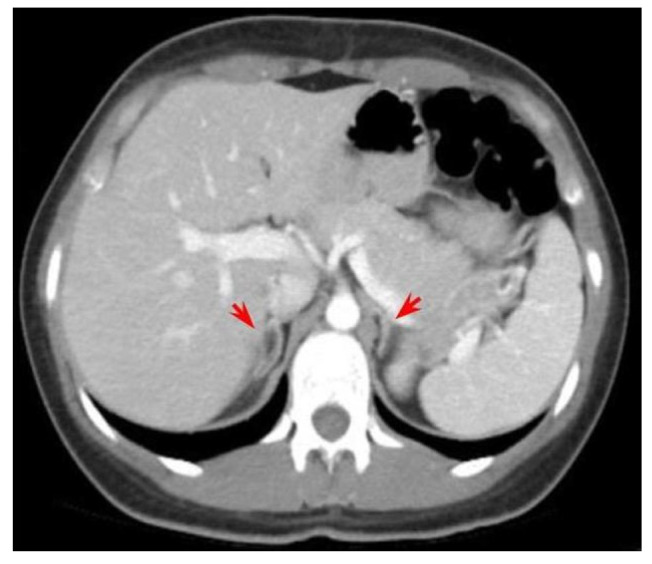
Bilateral adrenal hyperplasia (red arrows) according to computed tomography exam on a prior unreported case of a 26-year-old female diagnosed with HSD3B2 deficiency and SV-CAH form (no tumour was suspected in this case since the imaging aspect is rather usual in this situation).

Another subject of controversy might be claimed by continuing changes of the terminology and classifications in the area of adrenal tumours [112]. Our decade-based analysis included the original terms (from the publications), since it is difficult to adjust them to current names and this might come as a potential source of bias. As mentioned, we did not include CAH cases with typical adrenal hyperplasia at initial imaging assessment unless a tumour was particularly diagnosed (despite the fact that some post-adrenalectomy reports in fact showed an adrenal hyperplasia in some tumours, as they had been identified at computed tomography or magnetic resonance exams) [52,67] (Figure 3).

Finally, from our perspective, one of the greatest pitfalls of touching the topic of adrenal tumours in CAH is represented by addressing the term “adrenal incidentaloma”. Generally, an accidentally detected tumour at any organ subscribes to this name; however, the strictly endocrine perspective also means a negative endocrine profile and a slow growth rate (actually, the most common endocrine incidentaloma is a thyroid nodule, but the term is not usually applied to this gland in daily practice, but rather for adrenals and pituitary glands) [113,114]. Yet, as seen in mentioned studies, incidentalomas in CAH were not always harmless and the histological reports were heterogeneous, and thus the presence of an incidentaloma in CAH remains an open chapter and awareness of its particular significance is needed. Also, it might seem that CAH patients are more frequently assessed and thus it increases the risk of detecting an adrenal tumour, but, as already shown, many adrenal masses were found in poorly controlled and uncompliant cases; that is why a general conclusion is debatable. Moreover, our 10-year sample-based study included the COVID-19 pandemic period (between March 2020 and the first months of 2023); it is difficult to establish if fewer imaging investigations have been performed in these patients due to the medical and social regulations amid first waves. However, as seen in other medical and surgical domains, the pandemic impact should be noted when referring to the incidence of tumours, including adrenal incidentalomas [115,116,117,118,119,120].

### 4.3. A Matter of Surgery or Surgery Matters

Most patients were managed surgically (38/59), especially those with the largest tumours and the symptomatic subjects. The question of whether adrenalectomy may be postponed remains open, considering the fact that the majority of the adrenal masses were myelolipomas, slow-growing, benign tumours that had clear characteristics on computed tomography imaging. However, the gigantic sizes of up to 40 cm indicate that the subjects should be monitored to identify and remove in time such large tumours, which can possibly cause compression of nearby organs, which indicates adrenalectomy [121]. Whether the adrenal removal will be performed via a traditional approach or the laparoscopic route (that is preferred nowadays) represents a strictly surgical decision based on prior endocrinological and imaging assessments, as generally seen in other areas of adrenal tumours [122,123].

When choosing surgery, it is important to know whether patients with adrenal tumours may suffer from CAH, due to the risk of developing adrenal insufficiency [59,77]. Sometimes, adrenal failure occurs years after (unilateral) surgery [77]. Currently, the decision of adrenal removal is based on an individual decision rather than a guideline indication. Falhammar et al. [124] showed in 2016 according to a meta-analysis (n = 36 articles) with respect to CAH or carrier status in patients with adrenal incidentalomas that bilateral tumours “were frequent in CAH” (but bilateral lesions did not predict the most frequent CAH deficiency, namely CYP21A2) [124]. Consequently, the decision of tumour removal is even more challenging in these cases.

### 4.4. Limits of the Topic and Further Expansion

We are aware of the potential bias that comes with a narrative review, but we intended to cover a heterogeneous panel of issues in the area of adrenal tumours and CAH and not restrict the research. Also, the level of statistical significance of the cited papers varied from a single case report to studies with large sample sizes. Whether disease control or high ACTH levels are contributors to the development of such adrenal tumours in CAH is yet to be explored as a potential pathogenic link. Another potential bias may be the fact that the incidental detection of a tumour in a patient diagnosed with CAH does not necessarily mean causality, only a co-existence and that further studies are necessary. Also, the largest tumours are usually referred to adrenalectomy, and thus a potential bias with respect to the histological report of the entire panel of different tumours is expected. As mentioned, the term “incidentalomas” is not associated with any pathological report, and that is why the exact imaging-histological picture is difficult to assess in the absence of surgery. Another open issue remains the selection of adrenalectomy candidates and the exact protocol of imaging surveillance in CAH.

## 5. Conclusions

This 10-year, sample-based analysis represents one of the most complex analyses in the area of adrenal tumours in CAH so far. These masses should be taken into consideration for various reasons. They may reach impressive sizes of up to 30–40 cm, sometimes leading to symptoms such as abdominal pain and urinary tract obstruction. In these cases, adrenalectomy is the preferred management. Many such tumours are actually detected in subjects with poor disease control, or they represent the first step toward CAH identification. We noted a left lateralization with a pathogenic explanation that is less clear at the present time. The most frequent tumour remains myelolipoma. However, the risk of adrenocortical carcinoma should not be overlooked. Moreover, with the increasing prevalence of adrenal incidentalomas, CAH testing might be indicated, including to identify NC-CAH forms to provide adequate management.

## Figures and Tables

**Table 1:** Case reports (n = 13)/series (n = 5) with data regarding the clinical features of the patients diagnosed with CAH (CYP21A2 deficiency) and adrenal tumours/masses according to our strategy; the display starts with the earliest publication date within the decade of analysis [50,51,52,53,54,55,56,57,58,59,60,61,62,63,64,65,66,67].

First Author/Reference/Year	Studied Population/Age (Years)/Gender		CAH Form	Age at CAH Diagnosis (Years)	Age at Tumour Diagnosis (Years)
Almeida [50] 2014	N = 4 with ML (2/4 with SV-CAH)	35/F	SV	<20	35
		52/F	SV	NA	52
Falhammar [51] 2014	N = 9 (5/9 with genetic confirmation)	88/F	NC	88	88
		48/F	NC	48	48
		21/F	NC	21	21
		41/F	carrier	41	41
		67/M	SV	67	67
Libé [52] 2014	77/M		NC	77	77
Falhammar [53] 2016	42/M		NC	42	42
Kocak [54] 2016	46/M		NC	43	46
Buitenwerf [55] 2017	43/M		SV	43	43
Feng [56] 2017	61/M		SV	61	61
Hui [57] 2017	65/phenotypically male (46,XX karyotype)		SV	65	65
Mallappa [58] 2017	29/F		SV	29	29
Hirai [59] 2018	71/M		NC	71	49
Liu [60] 2018	N = 5 with ML	59/M	NC	59	46
		his brother	NC	NA	50
Kim [61] 2019	64/M		SV	64	56
Suchartlikitwong [62] 2019	39/M (46,XX karyotype)		SV	39	39
Aveiro-Lavrador [63] 2021	37/M		SV	37	35
San Martín [64] 2021	N = 4 with adrenal tumours (out of 8 males with CAH)	21/M	SW	NA	21
		23/M	SV	3 months	23
		28/M	SV	28	28
Burman [65] 2021	31/F		SW	at birth	28
Robinson [66] 2022	48/M		SV	48	48
Tang [67] 2023	N = 3 with CAH and 2 with adrenal tumours	37/M	NC	37	37
		59/M	NC	59	59

Abbreviations: CAH = congenital adrenal hyperplasia; F = female; M = male; ML = myelolipomas; N = number of patients; NC = non-classical; NA = not available; SW = salt-wasting; SV = simple virilizing.

**Table 2:** Case reports/series introducing adrenal tumours identified in patients with genetically confirmed *CYP21A2* deficiency according to our methods (the display starts with the earliest publication date) [50,51,52,53,54,55,56,57,58,59,60,61,62,63,64,65,66,67].

Reference	Tumour: Uni/Bilateral; Size (cm); Site (Left/Right)	Type	Clinical Presentation → Treatment
[50]	BAT: 14 × 14 × 10 cm (L) + 8.9 × 8.3 × 8.0 cm (R)	ML	Abdominal pain → adrenalectomy
	BAT: 16 × 13 × 9.0 cm (L) + 5.3 × 4.3 × 6.9 cm (R)	ML	Abdominal pain → adrenalectomy
[51]	LAT: 3 × 4 × 5 cm	I	Abdominal pain, constipation → prednisolone 5 mg/day
	two LAT: 1.3 × 1 cm + 1.4 × 1.5 cm	I	CT performed due to pneumonia; oligomenorrhea (PCOS) → prednisolone 5 mg (when needed)
	LAT: 1 cm	I	Diffuse abdominal discomfort (PCOS)→ patient declined any treatment
	BAT: 1 cm (L) + 0.9 cm (R)	I	Hirsutism, fertility issues (PCOS), abdominal discomfort → lifestyle changes (carrier)
	RAT: 8 cm	I	Abdominal discomfort → right adrenalectomy (benign)
[52]	LAT: 2 cm	ACC	Gynecomastia → adrenalectomy (causality cannot be established) → ↗ post-operatory 17OHP
[53]	LAT: 5.5 cm × 3.6 cm × 4.5 cm	I	Asymptomatic → hydrocortisone (at stress)
[54]	BAT: 10.6 × 7.0 × 5.5 cm (L) + 8.1 × 4.2 x3.3 cm (R)	ML	Palpable mass → bilateral adrenalectomy
[55]	LAT: 5.2 × 4.4 cm	I	Fatigue and myalgia → dexamethasone 0.5 mg/day
[56]	BAT: 18.2 × 16.2 × 14 cm (L) + 6.4 × 8.7 × 7.8 cm (R)	ML	Short stature, azoospermia, precocious puberty, increased testosterone in spite of anti-androgenic treatment for prostate cancer → glucocorticoid replacement
[57]	RAT:5.8 × 3.9 × 4.5 cm	HMA	Lower urinary tract symptoms, empty scrotum, small penile length, short stature → right adrenalectomy (the patient also had small uterine-like structure, small ovaries and prostate)
[58]	LAT: 10 × 7 cm	ML	Hyperpigmentation, abdominal pain, kidney compression → left adrenalectomy
[59]	LAT: 3–4 cm	I (ACA)	Left adrenalectomy at 49 years of age → adrenal insufficiency 22 years after adrenalectomy
[60]	giant BAT (R > L)	ML	Abdominal pain → bilateral adrenalectomy → 1.56 kg (L) and 3.05 kg (R)
	giant BAT	ML	Bilateral adrenalectomy
[61]	LAT: 12.5 × 7.5 cm RAT: diffuse nodular enlargement	ML	Adrenal hyperplasia identified on CT, early puberty → left adrenalectomy at 56 years of age
[62]	BAT: 2.3 × 1.4 × 2.5 cm (L) + 6 × 4.5 × 5cm (R)	ML	Adrenal insufficiency → glucocorticoid replacement ML were diagnosed based on CT and MRI findings
[63]	ML: 3 cm (R); ML: 2 cm (L)	ML + EACA	Consanguinity; personal history of precocious puberty, infertility, back pain → right adrenalectomy
[64]	RAT: 0.6 cm × 1.2 cm	NA	Three patients had genetic testing, while one patient did not
	LAT: 1.5 cm × 1.7 cm	NA	NA
	RAT: 4.9 cm × 3.4 cm	I (ACA)	Mass incidentally found during work-up for biliary colic → surgery
[65]	BAT: 11 cm (R) + 11 × 8.5 × 13 cm (L)	ML	RAT growth → right adrenalectomy → left adrenalectomy after 7 years
[66]	BAT: 8.4 × 6.2 × 7.5 cm (L) + 1.6 × 2 × 2.5 cm + 1.7 × 1.5 × 1.8 cm (R)	I (ML)	Short stature → left adrenalectomy and distal pancreatectomy → hydrocortisone therapy
[67]	RAT: 4.3 × 3.7 cm	AH	Azoospermia, small testes, hypospadias, premature pubarche, short stature → replacement therapy (the patient also had secondary hypogonadotropic hypogonadism)
	BAT: 20 × 25 cm (L) and 30 × 40 cm (R)	ML	Abdominal distension, surgical resection → glucocorticoid and mineralocorticoid replacement (the patient’s brother also underwent surgery for bilateral giant MLs at the age of 50)

Abbreviations: ACA = adrenocortical adenoma; ACC = adrenocortical carcinoma; AH = adenomatous hyperplasia; BAT = bilateral adrenal tumour; CT = computed tomography; EACA = ectopic adrenocortical adenoma; HMA = hyperplasia with mild atypia; I = incidentaloma; LAT = left adrenal tumours; L = left; ML = myelolipoma; MRI = magnetic resonance imaging; NA = not available; PCOS = polycystic ovary syndrome; RAT = right adrenal tumour; R = right; ↗ = increase.

**Table 3:** Gene testing results in case reports/series confirmed with *CYP21A2* deficiency and adrenal tumours/masses (the display starts with the earliest publication date) [50,51,52,53,54,55,56,57,58,59,60,61,62,63,64,65,66,67].

Reference	Genetic Testing Results
[50]	Compound heterozygote p.E351V, p.I236N, p.V237E, and p.M239K genetic variants in the *CYP21A2* gene
	IVS2-13A/C>G/p.I172N
[51]	V281L and I172N
	V281L
	V281L/V281L
	I172N
	I172N/deletion
[52]	Biallelic micro-conversion between the promoter regions of *CYP21A2* and the pseudogene *CYP21A1*
[53]	Compound heterozygous genetic variants: Pro30Leu genetic variant on one allele and a novel heterozygous duplication (c.264_276dup (p.Glu93Cysfs*5)) on the second allele
[54]	Homozygous g.656A/C>G point genetic variant
[55]	Compound heterozygous genetic variants: c.518T>A (p.Ile173Asn) and c.710T>A, c.713T>A, c.719T>A (p.lIe237Asn), (p.Val238Glu), (p.Met240Lys)
[56]	Complete gene deletion on one allele and a C518T>A (I172N) genetic variant on the other
[57]	Compound heterozygous p.Ile172Asn, p.Arg483Pro, and p.Met485Trpfs*56 genetic variants
[58]	Heterozygosity for intron 2 IVS2-13A/C>G splice site genetic variant/p.R483P (c.1451_1452 deletion insertion of C)
[59]	Micro genetic variant I172N and heterozygous large gene deletion or conversion
[60]	Compound heterozygous genetic variant: c.293-13C>G and c.518T>A, p.I173N
	Compound heterozygous genetic variant: c.293-13C>G and c.518T>A, p.I173N
[61]	IVS2-13A/C>G and p.I173N
[62]	Compound heterozygous R356W and intron 2G genetic variant
[63]	Variant g.655C>G
[64]	Heterozygosity: p.Gln318Ter (Q318) and p.Gly110ValfsTer21 (Del8bpE3)
	Homozygous genetic variant c.293-13C>G
	p.Ile172Asn (p I172N) and Del/Conv
[65]	Gln318stop/deletion in the *CYP21A2* gene
[66]	c.293–13C>G genetic variant on both alleles
[67]	Compound heterozygous genetic variant c.293-13C>G and c.518T>A, p.I173N
	Compound heterozygous genetic variant c.293-13C>G and c.518T>A, p.I173N

**Table 4:** Data regarding tumour size and hormonal findings at presentation in patients with genetically confirmed *CYP21A2* deficiency [50,51,52,53,54,55,56,57,58,59,60,61,62,63,64,65,66,67].

Reference	Tumour Size (cm)	Hormonal Panel at Presentation for Adrenal Tumour		
		ACTH	17-hydroxyprogesterone	Other Hormonal Assays or Observations
		(pg/mL)	(ng/mL or nmol/L)	
[50]	>10	1172	192 ng/mL	Testosterone = 949 ng/dL; Androstendione = 17 ng/mL
	>10	NA	120 ng/mL	Testosterone = 720 ng/dL; Androstendione = 39 ng/mL
[51]	5–10	NA	NA	Late diagnosis
	<5	NA	37 nmol/L	
	<5	NA	32 nmol/L	
	<5	NA	11.1 nmol/L	
	5–10	NA	338 nmol/L	
[52]	<5	NA	42 nmol/L	Late diagnosis
[53]	5–10	normal	51 nmol/L	Patient received diagnosis after incidentaloma was discovered
[54]	>10	214	28.6 ng/mL	DHEA-S = 29 (N:80-560) μg/dL
[55]	5–10	27	426 nmol/L	Testosterone = 13 nmol/L; Androstendione = 14 nmol/L
[56]	>10	NA	NA	Late diagnosis
[57]	5–10	NA	markedly raised	Testosterone normal (late diagnosis)
[58]	5–10	NA	17,900 ng/dL	Late diagnosis
[59]	<5	1820	9.4 ng/mL	Testosterone = 3.26 ng/mL; Androstendione = 1.15 ng/mL
				Late diagnosis, poor compliance with treatment
[61]	>10	157.6	27,500 ng/dL	Late diagnosis
[62]	5–10	10,445	2003 ng/dL	Late diagnosis
[63]	<5	1351	57 ng/mL	Testosterone = 0.7 ng/mL; Androstenedione = 4.5 µg/mL; late diagnosis
[64]	<5	NA	NA	
	<5	NA	NA	Irregular adherence to treatment during childhood
	<5	NA	NA	
[65]	>10	3	269 nmol/L	Androstenedione and 17-hydroxyprogesterone with fluctuant pattern
[66]	5–10	160	6078 ng/dL	Testosterone = 447.0 ng/dL; DHEA-S = 598 ug/dL; late diagnosis
[67]	<5	1131	485.20 nmol/L	Testosterone = 4.05 (normal: 1.75–7.81) ng/mL; DHEA = 7.99 ng/mL; late diagnosis
	>10	NA	NA	Late diagnosis

Abbreviations: ACTH = Adrenocorticotropic Hormone; DHEA-S = dehydroepiandrosterone-sulfate; NA = not available.

**Table 5:** Clinical and tumour features and associated outcome in patients with CYP17A1 deficiency; the display starts with the earliest publication date between 2015 and 2023, according to our methods [60,68,69,70].

First Author/Reference/Year	Patient Age/Gender		Genetic Testing (CYP17A1 Deficiency)	Age at CAH Diagnosis /Tumour Diagnosis (Years)	Tumour Features	Clinical Features	Therapy
Lee [68] 2015	36/F		Compound heterozygous genetic variant for p.Tyr329fs (c.985_987delTACinsAA) + missense genetic variant p.His373Leu (c.1118A > T)	36/36	10 × 6.3 × 8.6 cm Adrenal cortical adenoma	Abdominal pain Tanner 1	Left adrenalectomy
Liu [60] 2018		Patient 3: 36/F	Compound heterozygous genetic variant: c.1118A>T, p.H373L, and c.1459_1467del9, p.D487_F489del	36/36	Giant bilateral adrenal masses, the largest (left) of 20 cm × 15 cm × 10 cm Myelolipoma	Headaches, hypokalaemia since childhood, +hypertension (prior 6 years)	Left and right adrenalectomies
		Patient 4: 32/F	Compound heterozygous genetic variant: c.1118A>T, p.H373L, and c.1459_1467del9, p.D487_F489del	32/32	Myelolipoma	Fatigue, hypokalaemia	Left adrenalectomy
		Patient 5: 37/F	Compound heterozygous genetic variant: c.1118A>T, p.H373L, and c.1459_1467del9, p.D487_F489del	37/37	Myelolipoma	Hypertension, hypokalaemia (3 years prior)	Left and right adrenalectomies
Yang [69] 2019	27/F (46,XX karyotype)		Compound heterozygous genetic variant: c.985_987delTACinsAA (p.Tyr329fs) in exon 6 (frame-shift genetic variant) and c.1270C>T (p.Gln424) in exon 8 (nonsense genetic variant)	27/27	Myelolipoma	Persistent hypokalaemia, primary amenorrhoea	Dexamethasone 0.75 mg/day
Chang [70] 2023	31/F (46,XY karyotype)		Heterozygous variant of c.985_987delinsAA (p.Y329Kfs*90) and p.R96W genetic variant	31/31	Left adrenal mass of 5 × 9 cm myelolipoma	Hypokalaemia, hypertension, primary amenorrhea, hypoplastic breasts, vaginal infantilism, lack of axillary and pubic hair	Surgical treatment of the adrenal myelolipoma and resection of gonads

Abbreviations: F = female; NA = not available.

**Table 6:** Clinical and tumour characteristics in reports with CYP11B1 and HSD3B2 defects [71,72].

First Author Reference Number Year of Publication	Type of Study	Patient		Gene-Enzyme Deficiency	Gene Testing	Age at CAH Diagnosis (Years)	Age at Tumour Diagnosis (Years)
Ozbas [71] 2023	Case report	35-year-old female (C1)		CYP11B1	Homozygous missense genetic variant (c.1385T >C L462P variant (NM_000497.3)	35	35
Ladjouze [72] 2022	Mixed longitudinal and cross-sectional study	14 patients from 10 families with HSD3B2 deficiency	16-year-old female (C2)	HSD3B2	p.(Pro222GIn)	14 days	16
			13-year-old female (C3)	HSD3B2	p.(Thr152_Pro155del)	3 months	13
			15-year-old female (C4)	HSD3B2	p.(Thr152_Pro155del)	4 weeks	15
**Tumour features and outcome**							
**Reference**	**Patient**	**Tumour size**	**Pathological report**	**Clinical presentation**	**Surgery**	**Observations**	
[71]	C1	Left adrenal mass of 7.4 × 5.5 cm	Myelolipoma	Hypertension, adrenal mass, clitoromegaly, deep voice, hirsutism, hypokalaemia history of 2 genital reconstruction during childhood	Left adrenalectomy	The patient was prescribed glucocorticoids during childhood, but did not follow it regularly (for the previous 5 years the patient did not take glucocorticoids)	
[72]	C2	Right adrenal mass of 2.7 × 3 cm	Adrenocortical hyperplasia	NA	Adrenalectomy	Initially, misdiagnosed as 21OHD mass had high suspicion of malignancy (unconfirmed)	
	C3 *	Left adrenal mass of 6.3 × 5.2 × 5.1 cm	Adrenocortical hyperplasia	NA	Adrenalectomy		

Abbreviations: C = case; NA = not available, 21OHD = 21-hydroxylase deficiency (* data on C4 were not specifically addressed).

**Table 7:** Hormonal panel according to the cases reported with CYP17A1, CYP11B1, and HSD3B2 deficiency at the moment of the adrenal tumour confirmation [61,68,69,70,71,72].

Reference	Hormonal Panel at Presentation for Adrenal Tumour		
	ACTH	Plasma Cortisol	Other Parameters
[68]	75.94 (normal: 7.2 to 63.6) pg/mL	NA	Na = 141 (normal: 135 to 145) mmol/L K = 3.3 (normal: 3.5 to 5.5) mmol/L Renin = 0.80 (normal: 1.31 to 3.95) ng/mL/hr Aldosterone = 183.31 (normal: 29.9 to 158.8) pg/mL Aldosterone to renin ratio = 22.9
[60]	>1250 pg/mL (↗)	<2 μg/dL (↘)	Hypokalaemia
	271 pg/mL (↗)	<2 μg/dL (↘)	Hypokalaemia
	503 pg/mL (↗)	<3.1 μg/dL (↘)	Hypokalaemia
[69]	41.56 (normal: 0–40) pg/mL	171.39 (normal: 268.94–579.39) nmol/L	Hypokalaemia (K = 2.1 mmol/L) 17OHP < 0.05 (0.05–1.02) ng/mL
[70]	225.80 (normal: 7.20–63.30) pg/mL	0.1 (normal: 50–250) ng/mL	Hypokalaemia (K = 2.10 mmol/L, normal = 3.50–5.30) mmol/L Plasma renin activity = 0.91 (normal: 4–38) pg/mL Aldosterone = 76.78 (normal: 40–310) pg/mL Estradiol = 1.4 (normal: 15–350) pg/mL Testosterone = 10.4 (normal: 80–600) pg/mL DHEA-S = 0.9 (normal: 830–3770) ng/mL
[71]	279 (normal: 0–246) pg/mL	4.8 (normal: 6.2–18) μg/dL	K = 2.7 mmol/L, Na = 140 mmol/L Androstenedione>10 (normal: 0.3–3.3) ng/dL Total testosterone = 236 (normal: 6–82) ng/dL 17OHP = 8.31 (normal: 0.2–1) ng/dL
[72]	NA	NA	17OHP = 2.14 (normal: 0.48–1.87) nmol/L 17OHPreg = 93 (normal: 0.13–13.7) nmol/L (one patient)

Abbreviations: DHEA-S = dehydroepiandrosterone-sulfate; K = potassium; Na = sodium; 170HP = 17-hydroxyprogesterone; 170HPreg = 17-hydroxypregnenolone; NA = not available; ↘ = decrease; ↗ = increase.

**Table 8:** General characteristics of the patients confirmed with CAH according to the hormonal panel (but not genetic testing confirmation) and of the adrenal tumours according to the mentioned methods [51,73,74,75,76,77,78,79,80,81,82,83,84,85,86,87,88,89].

First Author/Reference	Population	CAH Form	Method of CAH Diagnosis	Age at CAH Diagnosis	Age at Tumour Diagnosis
Al-Bahri [73]	39-year-old male	NA	Unclear	Childhood	39
Alvarez [74]	44-year-old female	SV	Virilizing CAH	Infancy/childhood	44
Kale [75]	51-year-old male	SW	NA	Infancy	51
Altieri [76]	42-year-old male	SV	Precocious puberty	4 years	42
Meng [77]	40-year-old male	NC	Hormonal work-up	40	31
Łebek-Szatańska [78]	32-year-old male	SW	Salt waste	Neonate	30
Piskinpasa [79]	41-year-old male	SV	ACTH stimulation test	41	41
Lim [80]	58-year-old male	SV	Hormonal + ACTH stimulation test	58	58
Pakalniskis [81]	61-year-old male	SV	NA	NA (known CAH)	61
Khalil [82]	27-year-old phenotypic male, 46,XX karyotype	SV	NA	Late childhood	27
Kienitz [83]	50-year-old male	SW	Salt waste	Childhood	50
San Martín [65]	42-year-old male	SV		Infancy	42
Falhammar [51]	56-year-old male	NC	ACTH stimulation test	56	56
	66-year-old male	NC	ACTH stimulation test	66	66
	48-year-old female	NC	ACTH stimulation test	48	48
	53-year-old female	carrier	ACTH stimulation test	53	53
Vemula [84]	68-year-old female	NC	Clinically and biochemically	68	68
Longoria-Dubocq [85]	36-year-old male	NA	NA	NA (known CAH)	36
La [86]	37-year-old born female with ambiguous genitalia identifying as male	SW	Salt waste	Neonate	27
Lin [87]	36-year-old male	SW		At birth	36
Jacobson [88]	49-year-old male	SW	Salt waste	Infancy	49
Soveid [89]	26-year-old female, XY karyotype		Hormonal work-up following hypertension diagnosis	26	26

Abbreviations: ACTH = Adrenocorticotropic Hormone; CAH = congenital adrenal hyperplasia; NA = not available; NC = non-classical; SV = simple virilizing; SW = salt-wasting.

**Table 9:** Adrenal tumour panel, clinical presentation, and hormonal profile at the moment of tumour identification in patients diagnosed with CAH (with no genetic confirmation) [51,73,74,75,76,77,78,79,80,81,82,83,84,85,86,87,88,89].

Reference/ Age/Sex/ Enzyme Defect/ CAH Form	Tumour → Decision of Surgery (If Any)	Clinical Picture	Hormonal Panel at the Moment of Tumour Evaluation + Other Highlights
[73] 39/M CYP21A2 NA	BAT: 20 × 20 × 25 cm (L) + 16 × 12 × 15 cm (R) → ↗ size despite therapy → bilateral adrenalectomy	Abdominal distension, discomfort	ACTH = 42 (normal:6–50) pg/mL 17OHPg = 14,076 (normal: 42–196) ng/dL Testosterone = 506 (normal: 241–827) ng/dL
[74] 44/F CYP21A2 SV-CAH	26 × 24 × 9.5 cm (L) → surgery (exploratory laparotomy and mass excision)	Abdominal distension, nausea, vomiting	NA
[75] 51/M CYP21A2 SW-CAH	BAT: 31.1 × 18.1 × 16.1 cm (L) + 13.7 × 6.6 × 10.6 cm (R) → bilateral adrenalectomy	Chronic back pain, lower limbs parasthesiasis	The patient was under long-term supra physiological glucocorticoid replacement, without biochemical monitoring
[76] 42/M CYP21A2 SV-CAH	BAT: 16 × 13 × 9.0 cm (L) + 5.3 × 4.3 × 6.9 cm (R) → left adrenalectomy	Recurrent abdominal pain, digestive symptoms	Despite right tumour growth, the patient remained asymptomatic and denied a second surgical intervention
[77] 40/M CYP21A2 NC-CAH	5 × 4 cm (R) 4.1 × 3.9 cm (L) (diagnosis: 9 years after right adrenalectomy)	Adrenal insufficiency after initial right adrenalectomy	ACTH > 2000 (normal: 5.0–78) pg/mL 17OHPg = 21.13 (normal: 0.31–2.01) ng/mL Testosterone = 1.81 (normal: 2.49–8.36) ng/mL Cortisol = 157.8 (normal: 147.3–609.3) nmol/L
[78] 32/M CYP21A2 SW-CAH	BAT: 6.7 × 4.8 × 2.7 cm (R) + 19.8 × 19.1 × 12 cm (L) → left adrenalectomy → adrenal carcinoma → mitotane (The patient was awaiting for the right adrenalectomy)	Rapidly enlarging BAT	NA
[79] 41/M CYP21A2 SV-CAH	BAT: 4.1 × 2.2 cm (L) + 8.8 × 5.5 cm (R) (incidentally detected during follow-up of testes tumours) → right adrenalectomy → myelolipoma	Incidental imaging diagnosis	ACTH = 80.4 (normal: 9–46) pg/mL 17OHPg = 14 (normal: 0.2–2.3) ng/mL Testosterone = 1.79 (normal: 2.18–9.06) ng/mL Cortisol = 3.75 (normal: 6.2–19.4) μg/dL
[80] 58/M CYP21A2 SV-CAH	10 cm (L) → planned surgery		ACTH = 181 (normal: 0–60) pg/mL 17OHPg = 13,800 (normal: 20–172) ng/dL Testosterone = 6.75 (normal: 2.5–10.63) ng/mL Cortisol = 8.6 (normal: 9.4–26.1) μg/dL
[81] 61/M CYP21A2 SV-CAH	10.3 cm (R) with calcifications + 2.9 cm (L) suggestive of myelolipoma → right adrenalectomy	Pressor-dependent shock	High ACTH High 17OHPg The patient had Mullerian structures (prior known with pseudo hermaphroditism
[82] 27/M * CYP21A2 SV-CAH	9 × 8 × 7 cm (L) with calcifications and central necrosis measuring (of 5.5 cm) → left adrenalectomy	Incidentaloma on abdominal ultrasound	Normal ACTH High 17OHPg
[83] 50/M CYP21A2 SW-CAH	BAT with septic lobular appearance of 10 × 11 × 6 cm (L) + 14 × 19 × 11 cm (R) → right adrenalectomy	Polakidisuria	ACTH = 37.5 (normal: 1.6–45) pg/mL 17OHPg = 0.6 to 1.5 (normal: 0.2–1.4) ng/mL Testosterone = 1.7(normal:1.3–7.7) ng/mL Cortisol = 0.24 to 0.5 (normal: 0.5–3.5 ng/mL)
[64] 42/M CYP21A2 SV-CAH	Bilateral thickening with left predominance <1.3 cm		NA
[51] 56/M CYP21A2 NC-CAH	BAT: 1.2 cm (L) + 0.9 cm (R)	Abdominal pain	17OHPg = 14 nmol/L
[51] 66/M CYP21A2 NC-CAH	BAT of 1.5 cm the largest		17OHPg = 3.4 nmol/L
[51] 48/F CYP21A2 NC-CAH	BAT: 1.5 × 2 cm (L) + “minor” tumours (R)	Abdominal discomfort	17OHPg = 6.9 nmol/L
[51] 53/F CYP21A2 carrier	BAT: 3.3 × 3 cm (L) + 1.3 × 3.3 (R)	Abdominal pain	17OHPg = 2.3 nmol/L
[84] 68/F CYP21A2 NC-CAH	BAT: 6.6 × 9.7 × 10.5 cm (L) + 3 × 7.6 × 6.8 cm (right) → myelolipoma diagnosis was based on CT findings	Chest discomfort, virilization, hirsutism, excessive labial folds	ACTH = 266.7 (normal: 7.2–63.3) pg/mL 17OHPg = 25,018 (normal: 15–70) ng/dL Testosterone = 1195 (normal: 60–80) ng/mL Cortisol ** = 2.8 (normal: <1.8) µg/dL
[85] 36/M NA NA	left retroperitoneal mass of 30 × 23.6 × 16.7 cm → tumour developed despite of adequate CAH management → tumour resection	Abdominal pain, difficulty breathing	NA
[86] 37/ *** NA SW-CAH	BAT: 11.8 × 8.8 cm (L) + 5.9 × 2.4 cm (R) → bilateral adrenalectomy & hysterectomy with bilateral salpingo-oophorectomy	Abdominal distension, hypotension, virilization	ACTH = 166 (normal: 6–50) pg/mL 17OHPg = 4356 (normal: 285) ng/dL Testosterone = 737 (normal: 2–45) ng/dL Cortisol = 78.5 (normal: 3.7–19.4) mg/dL
[87] 36/M NA SW-CAH	Adrenal mass of 23 cm (L) + adrenal nodule of 2.5 cm (R) → the tumours were incidentally found → adrenalectomy → glucocorticoid/mineralocorticoid treatment	Admission for dyspnoea (pulmonary embolism)	ACTH = 128 (normal: 6–50) pg/mL 17OHPg = 17,300 ng/dL Myelolipoma was diagnosed based on CT findings
[88] 39/M NA SW-CAH	BAT: 18 × 13.4 × 12cm (L) + 7.3 × 2.7 × 5.8 cm (R) → bilateral adrenalectomy due to abdominal pain	Salt craving, hyperpigmentation, small testes, abdominal pain	17OHPg = 8230 (normal < 220) ng/dL
[89] 26/F **** CYP17A1 hypertension	BAT: 6.5 cm (L) + 3 cm (R) → left adrenalectomy due to asymmetric enlargement and abdominal pain	Hypertension, Tanner 1	ACTH = 185 (normal: 6–76) pg/mL 17OHPg < 10 (normal: 20–100) ng/dL Testosterone = <0.02 (normal: 0.084–0.481) ng/mL Cortisol = 0.9 (normal: 5.4–28.7) μg/dL

Abbreviations: ACTH = Adrenocorticotropic Hormone; 17OHPg = 17-hydroxyprogesterone; BAT = bilateral adrenal tumours; CAH = congenital adrenal hyperplasia; CT = computed tomography; F = female; L = left; NA = not available; NC = non-classical; M = male; R = right; SV = simple virilizing; SW = salt-wasting (of note, “cortisol” means plasma morning cortisol); * = phenotypically male + 46,XX karyotype; **after 1 mg dexamethasone suppression test; *** born female with ambiguous genitalia identifying as male; **** phenotypically female + 46,XY karyotype.

**Table 10:** Prevalence studies of CAH among patients with adrenal tumours/incidentalomas or adrenal tumours amid CAH diagnosis; the display starts with the earliest publication date we could identify according to our strategy (between 2015 and 2023) [90,91,92,93,94,95,96,97,98].

First Author Reference Number/Year	Study Design	Study Population	Results
Askitis [90] 2015	Retrospective	187 patients with adrenal tumours, including 49-year-old male patient with CAH	Prevalence of CAH among adrenal tumours: 0.53%
Kiedrowicz [91] 2015	Retrospective	100 patients with AI, out of whom 8 had CAH	Prevalence of CAH genetic variants among AIs: 0.8%
Patrova [92] 2015	Retrospective	637 patients with AI, out of whom 2 had CAH	Prevalence of CAH among AIs: 0.3%
Kim [93] 2017	Retrospective	53 patients with CAH due to CYP21A2 deficiency	Long-term consequences of CAH due to CYP21A2 deficiency: prevalence of adrenal tumours among CAH: 0.9%
Goh [94] 2018	Prospective	228 patients with AI, out of whom 4 had CAH	Prevalence of CAH genetic variants among AIs: 1.8%
El-Maouche [95] 2019	Retrospective	88 patients with CAH	Myelolipoma prevalence among CAH:12.5%
Kim [96] 2022	Retrospective	90 adults with 21OHD and 270 healthy controls	CAH control and adrenal morphology → prevalence of adrenal tumour among CAH/21OHD: 13.3%
Sahlander [97] 2022	Prospective	320 individuals with AI, out of whom 8 had CAH	Prevalence of CAH among patients with adrenal incidentalomas: 3.6%
Sahlander [98] 2023	Retrospective (register-based)	26,573 individuals with adrenal tumours, out of whom 20 had CAH and 144,124 controls without adrenal tumours, out of whom 1 had CAH	Prevalence of CAH among patients with adrenal tumours: 0.75‰
Sahlander [98] 2023	Retrospective (register-based)	26,573 individuals with adrenal tumours, out of whom 20 had CAH and 144,124 controls without adrenal tumours, out of whom 1 had CAH	Prevalence of CAH among patients with adrenal tumours: 0.75‰

Abbreviations: AI = adrenal incidentaloma; CAH = congenital adrenal hyperplasia; 21OHD = 21-hydroxylase.

## Data Availability

Not applicable.

## Abbreviations

ACTH	Adrenocorticotropic Hormone
CAH	congenital adrenal hyperplasia
CYP21A2	21-hydroxylase
CYP17A1	17alpha-hydroxylase/17,20-lyase
CYP11B1	11-beta hydroxylase
HSD3B2	3-beta-hydroxysteroid dehydrogenase type II
CI	confidence interval
NC	non-classical
SV	simple virilizing
OR	odds ratio
SW	salt-wasting
